# Frontal and parietal background connectivity and their dynamic changes account for individual differences in the multisensory representation of peripersonal space

**DOI:** 10.1038/s41598-021-00048-5

**Published:** 2021-10-15

**Authors:** Sara Spadone, Mauro Gianni Perrucci, Giulio Di Cosmo, Marcello Costantini, Stefania Della Penna, Francesca Ferri

**Affiliations:** 1 Department of Neuroscience, Imaging and Clinical Sciences - and ITAB, Institute for Advanced Biomedical Technologies, G. d’Annunzio University of Chieti-Pescara, Chieti, Italy; 2 Department of Psychological, Health and Territorial Sciences - and ITAB, Institute for Advanced Biomedical Technologies, G. d’Annunzio University of Chieti-Pescara, Chieti, Italy

**Keywords:** Cognitive neuroscience, Sensorimotor processing

## Abstract

Functional connectivity (FC) of brain networks dynamically fluctuates during both rest and task execution. Individual differences in dynamic FC have been associated with several cognitive and behavioral traits. However, whether dynamic FC also contributes to sensorimotor representations guiding body-environment interactions, such as the representation of peripersonal space (PPS), is currently unknown. PPS is the space immediately surrounding the body and acts as a multisensory interface between the individual and the environment. We used an audio-tactile task with approaching sounds to map the individual PPS extension, and fMRI to estimate the background FC. Specifically, we analyzed FC values for each stimulus type (near and far space) and its across-trial variability. FC was evaluated between task-relevant nodes of two fronto-parietal networks (the Dorsal Attention Network, DAN, and the Fronto-Parietal Network, FPN) and a key PPS region in the premotor cortex (PM). PM was significantly connected to specific task-relevant nodes of the DAN and the FPN during the audio-tactile task, and FC was stronger while processing near space, as compared to far space. At the individual level, less PPS extension was associated with stronger premotor-parietal FC during processing of near space, while the across-trial variability of premotor-parietal and premotor-frontal FC was higher during the processing of far space. Notably, only across-trial FC variability captured the near-far modulation of space processing. Our findings indicate that PM connectivity with task-relevant frontal and parietal regions and its dynamic changes participate in the mechanisms that enable PPS representation, in agreement with the idea that neural variability plays a crucial role in plastic and dynamic sensorimotor representations.

## Introduction

Recent studies have revealed that the variability of brain activity is not simply “noise”; rather, it is orderly organized in a series of functional networks that maintain at all times a high level of spontaneous activity correlation^1^. A growing body of literature indicates that such functional connectivity (FC) dynamically fluctuates at time-scale of several seconds during rest^2^ and the execution of specific cognitive tasks (e.g.^3^). Importantly, dynamic FC has been associated with individual differences in a wide range of cognitive and behavioral traits, and it is often a more sensitive marker of such differences, compared to static FC^4^. In the present study, for the first time, we investigate the role of FC changes in the sensorimotor representation of peripersonal space (PPS), that is, the space immediately surrounding the body^5,6^.

The brain representation of PPS is extremely dynamic so that PPS extension dynamically adapts to mutable aspects of the context, either physical^7,8^ or socio-emotional^9–11^, and to changing action capabilities^12–18^. Moreover, the extension of PPS largely varies across individuals^19^. For instance, individuals with anxious^20^ or claustrophobic^21^ personalities show more extended PPS, while those with good interoceptive skills have less extended PPS^22^. Also, PPS extension adapts to the physical dimensions of the individual’s body (e.g., the arm length^23^). This complies with the model stating that sensorimotor plasticity and dynamic sensorimotor representations are enabled by neural variability^24,25^, which also allows adaptation to new environments^26^. Accordingly, we have recently shown that the intertrial variability in the premotor cortex, but not to the trial-averaged BOLD response in the same region, accounts for individual differences in PPS extension, as measured by an audio-tactile interaction task^19^. The premotor cortex (PM) is a key PPS brain region housing multisensory neurons (visuo-tactile, audio-tactile, visuo-audio-tactile) that preferentially respond to objects placed on, or near, the skin, especially if they dynamically approach the animal’s body^27–29^. PPS neurons are, instead, less sensitive to far and receding objects. However, PPS neurons have been identified also in the parietal cortices^27–29^ and, interestingly, frontal and parietal PPS regions in monkeys (e.g. F4 and VIP) seem to have direct and specific anatomo-functional connections^30^.

Consistently with neurophysiological studies in monkeys, neuroimaging studies on PPS in humans have reported several frontal and parietal regions that differentiate between near and far space (for a meta-analysis see^31^). Most of them (e.g. regions in the pre- and post- central gyrus, the anterior and dorsolateral prefrontal cortices, the inferior parietal cortex) overlap with nodes of two resting-state fronto-parietal networks, functionally known as Dorsal Attention Network (DAN) and Fronto-Parietal Network (FPN)^32–34^.

While localization of PPS regions has been extensively reported in monkeys and humans, the literature on functional connectivity between these regions and its contribution to the processing of near and far space in humans is scant. However, understanding how the frontal and parietal areas involved in PPS are functionally linked would be fundamental to unravel the mechanisms enabling the individual representation of near vs far space. To address this question, we examined the fMRI functional connectivity between a key PPS region (PM) and task-relevant nodes of the DAN and the FPN during the execution of a multisensory (auditory-tactile) task with approaching (looming) sounds designed to differentiate neural signals processing near and far stimuli^35^. We hypothesized that FC fluctuates during the processing of multisensory near and, especially, far stimuli given the dynamic and plastic character of PPS representation. Therefore, we analyzed the background time-varying functional connectivity (TVFC), computed with a fixed length sliding window approach^36,37^, to evaluate stimulus-specific connectivity and its variability across trials.

We expect to find significant background connectivity between PM and task-relevant frontal and parietal nodes of the DAN and the FPN during the multisensory PPS task. Moreover, we expect that the fluctuations of FC involving PM during the processing of near and/or far multisensory stimuli reflect individual differences in PPS extension.

## Results

### Background FC and individual PPS extension

**Figure 1:**
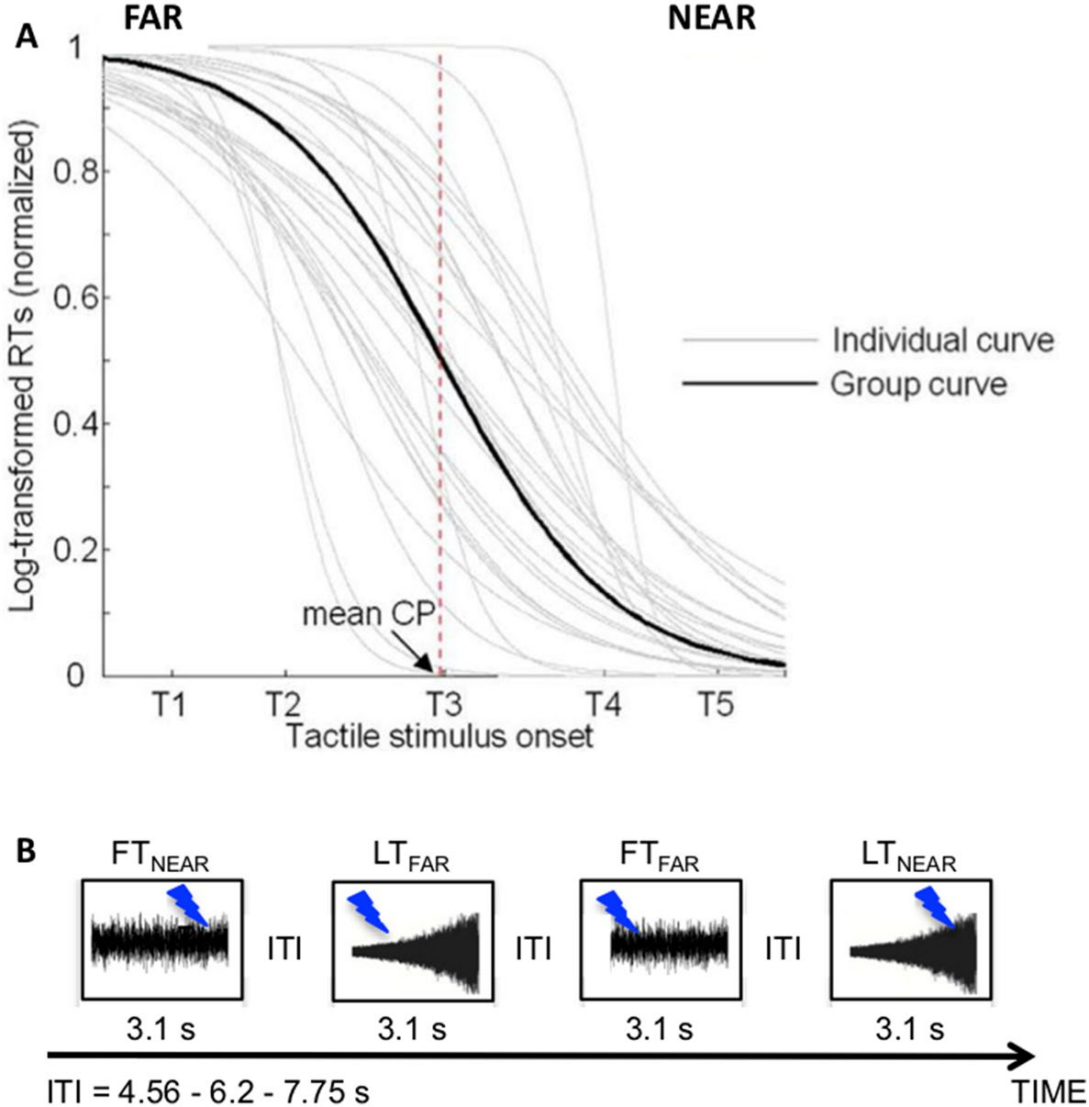
(**A**) Behavioral experiment: individual differences of PPS extension. Individual sigmoid fits obtained from log-transformed RTs to tactile target presented during looming sounds. Log transformation was applied using the natural log to correct the typical RT skew. The dashed vertical line identifies the group Central Point (CP) and corresponds to the critical distance at which looming sounds affected the participants’ tactile RTs. (**B**) fMRI experiment: design. Four experimental conditions were presented that resulted from the combination of the two types of sounds (looming, L and flat, F) and the two temporal delays of the tactile stimulus (TNEAR and TFAR). LTNEAR and LTFAR conditions allowed testing neural responses to multisensory events individually perceived as occurring either near or far, respectively. FTNEAR and FTFAR control conditions allowed obtaining BOLD responses to multisensory events at the same temporal delays, but not affected by the spatial information provided by sound intensity changes. ITI, Intertrial interval. Panel **A** is derived from results of our previous publication^19^.

**Figure 2:**
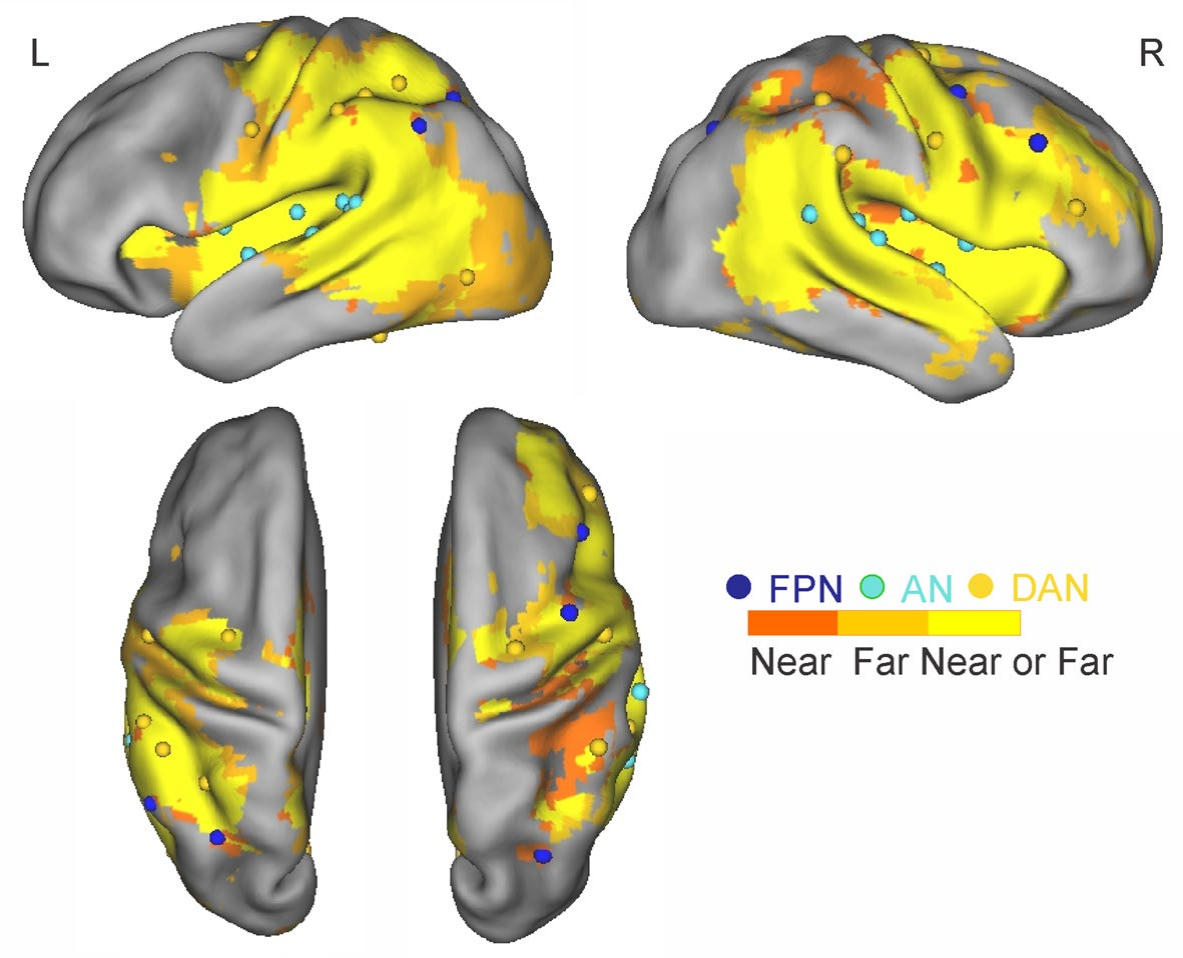
Voxels showing significant group-wise fMRI activation during near (dark orange), far (light orange) or during both space conditions (yellow) together with the representation of the ROIs selected from Baldassarre et al.^84^ superimposed over an inflated cortical representation. Different networks are color coded (blue: fronto-parietal network (FPN); cyan: auditory network (AN); orange: dorsal attention network (DAN)).

To identify the contribution of functional interactions to individual differences in PPS extension, 28 healthy volunteers participated in the present fMRI study, in which they performed an audio-tactile interaction task with approaching sounds designed to differentiate neural signals processing multisensory near and far stimuli. The boundary of peripersonal space, used to distinguish near from far space, was indexed by central point (CP) that was individually estimated through a behavioral session (“Materials and methods” and Fig. 1). We examined the background TVFC using the sliding window approach^37^ between PM, a key PPS region, and task-relevant nodes (Fig. 2) of the Dorsal Attention Network (DAN), the Fronto-Parietal Network (FPN) and the Auditory Network (AN), during near and far space processing.

**Figure 3:**
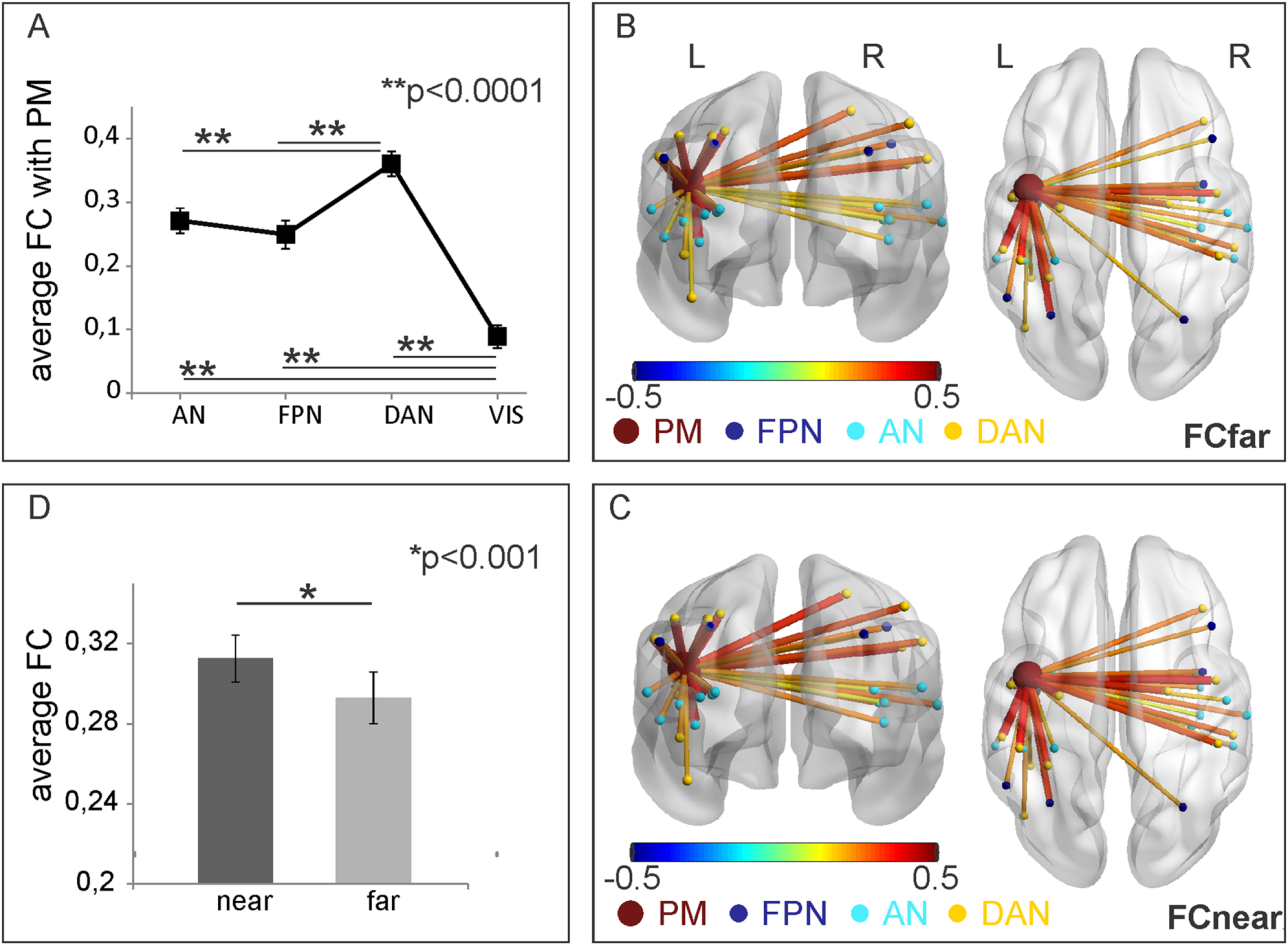
(**A**) Comparison among pairwise FCs between Premotor (PM) and task relevant regions (belonging to auditory network-AN, fronto-parietal network-FPN and dorsal attention network-DAN), and between Premotor and task irrelevant regions (belonging to visual network-VIS), as a control. (**B**–**D**) Results of background FC between PM and regions belonging to FPN, AN and DAN networks. Significant functional links are represented using lines on a standard brain in posterior and dorsal views. Line color and thickness represent the strength of connection during far (**B**) and near (**C**) space processing. (**D**) Average values of FC modulations across subjects and ROI pairs are shown as a bar plot together with the result of the two-sample t-test.

First, we compared the functional connections between PM and the task-relevant nodes (selected within DAN, FPN, AN) with the functional connections between PM and a set of task-irrelevant nodes belonging to the visual network (VIS), to test their specificity. To this aim, TVFC values were averaged over time and nodes in each Network, and entered into a one-way ANOVA, with Network (AN, FPN, VIS) as within-subject factor. Results revealed significant differences between networks (F_3,81_ = 82.2, *p* < 0.00001). Post-hoc comparisons revealed that PM-VIS FC was significantly lower than PM-AN, PM-FPN and PM-DAN FC (*ps* < 0.0001) regardless of space condition (Fig. 3A).

We then tested the statistical significance of PM-DAN, PM-FPN and PM-AN FC during near and far space processing (TVFC averaged over time for each stimulus type—see “Materials and methods”). The significant FC values during the two space conditions, as assessed by one-sample *t*-tests across subjects for all the ROI pairs (*p* < 0.05, Bonferroni corrected), are shown in Fig. 3B, C. The significant FCs are represented as lines on a standard brain (posterior and dorsal views) using a network visualization tool (BrainNet^38^). The thickness and the color of each line reflect the strength of the connectivity between two regions. Moreover, a direct comparison between FC during near and far stimuli using a two-sample *t*-test across pairs of ROIs revealed that the overall background FC was significantly stronger for near compared to far spatial condition (*p* < 0.001*;* Fig. 3D).

**Figure 4:**
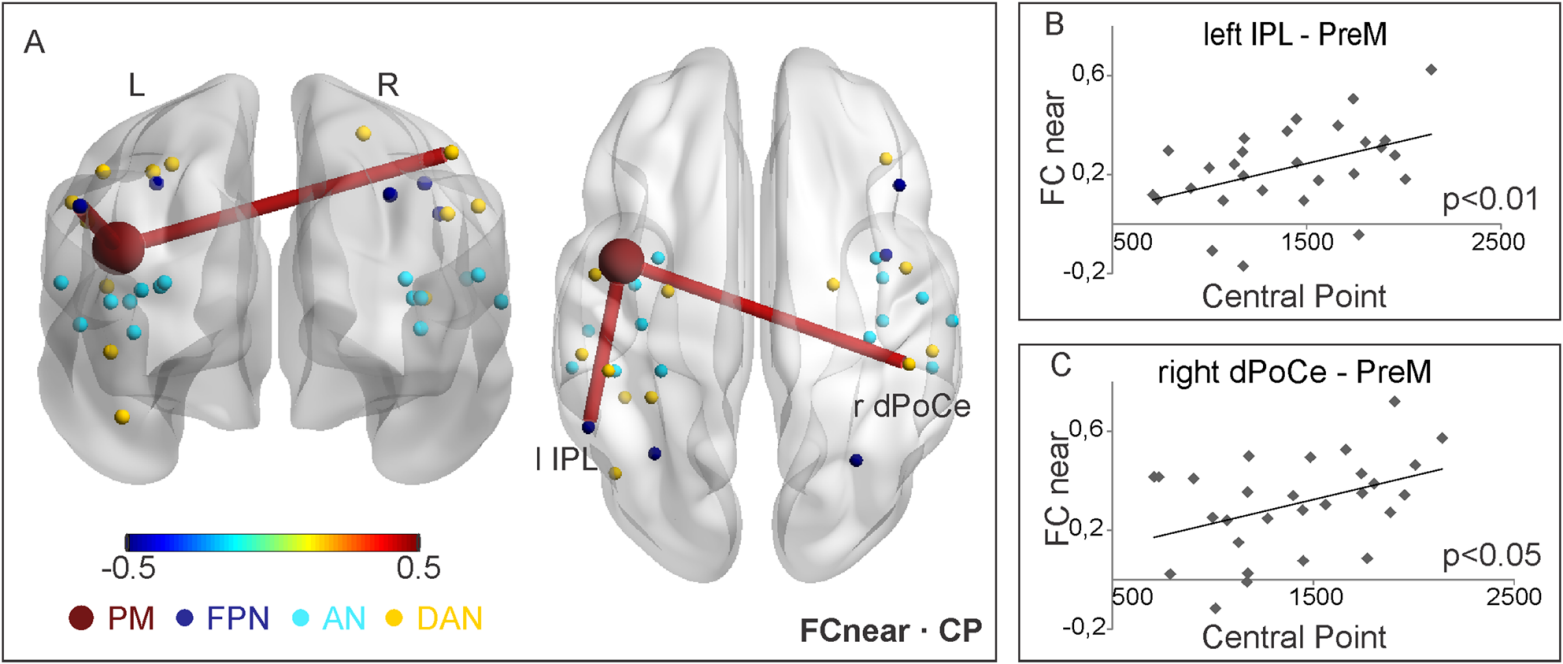
(**A**–**C**) Results of correlation between FC values and PPS extension (CP), during near condition. Significant correlation coefficients are represented as lines on the standard brain (**A**) and as scatter plots of FC as a function of CP (**B**, **C**).

We next tested whether the functional interactions between areas involved in the representation of PPS may contribute to individual differences in PPS extension. To this aim, for each node pair involving PM and a task-relevant node in the DAN, FPN and AN, we estimated the Pearson correlation coefficient between FC values (averaged over time for near and far stimuli) and individual extent of PPS (CP). The significant correlations for the near space condition (*p* < 0.05) are shown in Fig. 4A. The color and thickness of each line reflect the strength of the correlation. Importantly, only positive correlations between PM-left IPL connection and CP (r = 0.44; *p* < 0.01, Fig. 4B), and between PM-right dorsal PostCentral (dPoCe) connection and CP, (r = 0.41; *p* < 0.05, Fig. 4C) were statistically significant. In contrast, no significant correlations with CP were found in the far space condition (all *ps* > *0.05*).

Overall, our results suggest that, during the audio-tactile interaction task, the background functional connections between PM and task-relevant regions belonging to DAN, FPN and AN are significantly stronger than those observed between PM and a set of task-irrelevant nodes belonging to VIS. Also, these specific PM-DAN, PM-FPN and PM-AN links are generally stronger when subjects are involved in the processing of multisensory near events, as compared to far events. Finally, at the individual level, stronger FC between PM and both left IPL and right dPoCe, belonging to FPN and DAN respectively, are associated with larger CP (i.e., reduced PPS extension) during near space processing.

### Beta series correlation and individual PPS extension

We further tested whether the obtained relationships between FC and individual PPS extension could be found also using *β*-series correlations, which are based on trial-evoked BOLD responses, or if they were specific for the “background” brain signals, that is, independent from responses evoked at every single trial. To this aim, we first estimated the β-series correlations for the node pairs PM—left IPL and PM—right dPoCe, i.e. the links showing a significant correlation with CP, during the two space conditions. These were significant, as assessed by one one-sample *t*-test across subjects (left IPL: near and far r = 0.3; right dPoCe: near r = 0.32 and far r = 0.35; all *ps* < 0.0001). Then, we estimated the Pearson correlation coefficient between these *β*-series correlations during the near condition and CP. Interestingly, no significant effects were found for both PM-left IPL (r = 0.17; *p* > 0.05) and PM-right dPoCe (r = 0.06; *p* > 0.05).

### Variability of background FC and individual PPS extension

To identify whether the stability/variability of functional interactions is associated with individual differences in PPS, we first estimated the standard deviation of FC values across blocks of consecutive near and far trials (see “Materials and methods”), which is a measure of FC variability.

**Figure 5:**
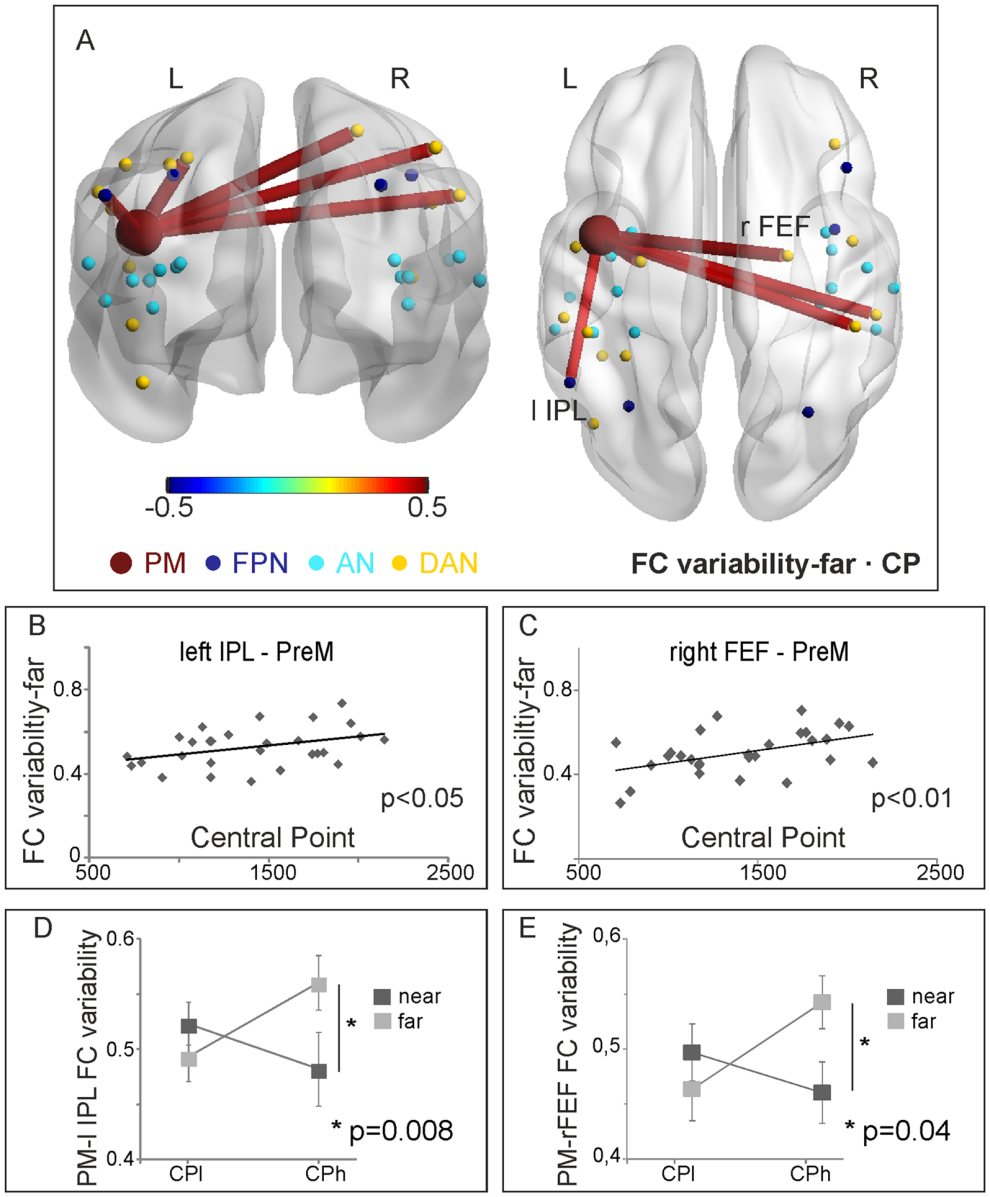
(**A**–**E**) Results of FC variability analysis. Significant correlation coefficient between FC variability and PPS extension (CP) during the far space condition is displayed on the standard brain in posterior and dorsal views (**A**) and using a scatter plot of FC variability as a function of CP (**B**, **C**). The variability of connectivity between PM and left IPL (**D**) and between PM and right FEF (**E**) is shown for two groups of subjects with different extension of PPS (participants with wide and narrow PPS, CPl and CPh respectively) in the near and far conditions. Significant interaction of group (CPh, CPl) x space (near, far), and the results of post-hoc test are shown in the corresponding panel.

We then estimated the Pearson correlation coefficient between stimulus-specific FC variability of the PM-DAN, PM-FPN and PM-AN links, limited to the task-relevant nodes, and individual CP. The obtained correlations for the far space condition are shown in Fig. 5A. For what concerns the FPN, only the correlation with FC variability between PM and left IPL was statistically significant (r = 0.38; *p* < 0.05, Fig. 5B). As for the DAN, statistically significant correlations were found with FC variability between PM and the following task-relevant nodes: the left dorsal PreCentral (left dPrCe; r = 0.46, *p* < 0.01), the right Frontal Eye Field (right FEF; r = 0.47, *p* < 0.01, Fig. 5C), the dorsal Post Central (right dPoCe; r = 0.42, *p* < 0.05) and the ventral Post Central—Supra Marginal Gyrus (right vPoCe-SMG; r = 0.43, *p* < 0.05). In contrast, correlations in the near space condition were not significant (all *ps* > 0.05). Given the functional significance of the BOLD signal variability in PM described in^19^, we ran the following test to rule out the possible association of the present with previous results. Specifically, we computed the correlation between the FC measures (strength and variability) and the trial-by-trial variability of the BOLD response (taken from our previous study^19^). No significant result was found for any of the links that showed a significant correlation with CP (− 0.24 < r < − 0.1; ps > 0.22).

**Figure 6:**
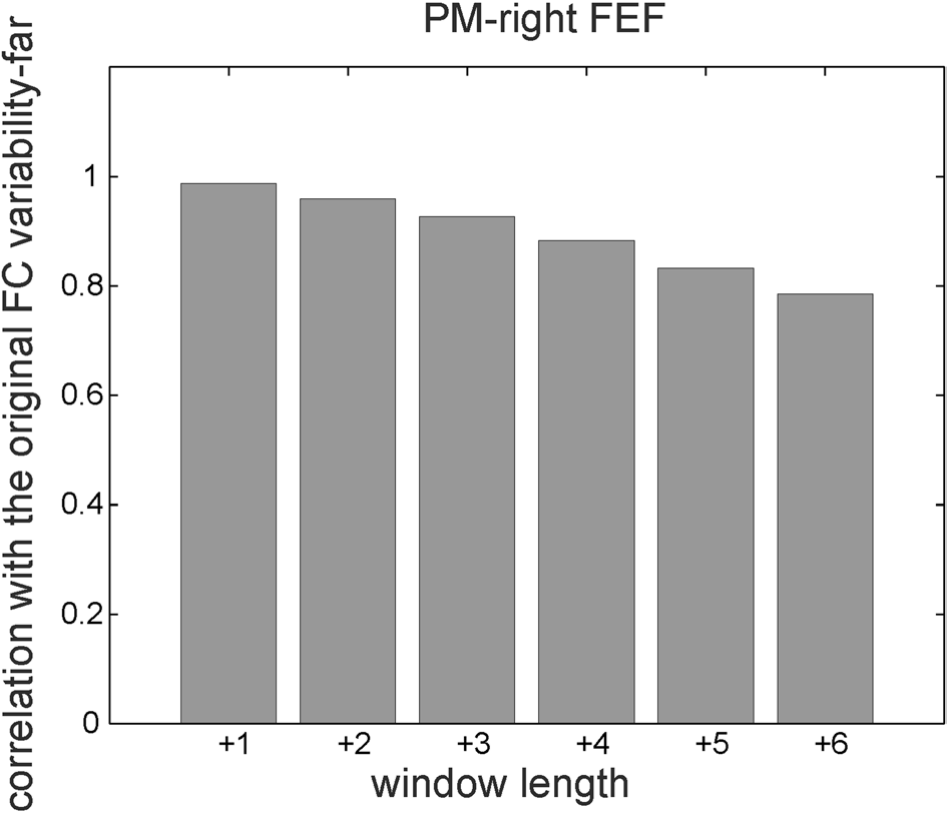
Correlation between FC variability and the one computed increasing the window length for PM-right FEF link.

Moreover, we performed additional control analyses to test the impact of factors that could influence the results obtained with TVFC estimated with the sliding-window technique. Specifically, it is well known that the window length used for the sliding window computation and the existence of autocorrelation within time series might impact the sampling variability of the estimator, which accounts for how much the TVFC varies from sample to sample^39^. The first set of control analyses tested the effect of the window length. In the present study, a short window length, which might inflate the FC variability, was required by the task design characterized by short blocks of consecutive near and far trials to prevent anticipation effects. Thus, we computed the time-varying Pearson coefficient between the PM region and all the ROIs over sliding windows stepped by 1 TR and increasing the window length (+ 1, + 2, + 3, + 4, + 5, + 6 points) with respect to the one we used for the main analysis. Then, we estimated the near and far FC variability obtained with the new window lengths. The FC variabilities were strongly correlated with the one from the main analysis (r > 0.9 up to + 3 and 0.8 < r < 0.9 up to + 6). Figure 6 shows the correlation between FC variabilities used for the main analysis and those obtained varying the size of the window for one sample link (PM to right FEF). Importantly, correlations between FC variabilities estimated for all the window lengths and individual CP were always statistically significant (ps < 0.05), thus, confirming our main results.

The second set of control analyses tested the effect of the autocorrelation present in the data that could inflate the sampling variability of TVFC estimated using the sliding-window technique^39^. From the original GLM residuals, we generated surrogated data using a phase randomization technique. First, we Fourier transformed the original data. Then, we randomly shuffled the Fourier phases series, and finally anti-Fourier-transformed back the data^40,41^. This provided surrogate data with the same autocorrelation function as the original timecourses. In this way, we obtained 500 replicates of the original correlation timecourses. Then, using the same approach as for the real data, we computed the null distribution of the correlation between across-trial FC variability during near and far conditions and CP. From this distribution, we estimated the threshold p-values to be applied to the main results. The significant links between FC variability and CP were confirmed using the comparison with the null distribution of correlation with CP obtained using surrogate data characterized by the same autocorrelation function. Specifically, the correlations between CP and PM-left IPL link and between CP and PM-ROIs belonging to the DAN were statistically significant (all ps < 0.01).

Finally, a significant potential source of artefactual nonstationarity, which can increase the variability in FC estimate, is the head motion^42^. Thus, eventual differences between TVFC measures from different trial blocks might be driven by systematic artifacts (this is even more likely for between-group comparisons). The influence of motion on TVFC was considered in the preprocessing step, specifically in the GLM by adding six parameters, obtained by rigid body head motion correction, in the regression. Therefore, the residual dataset minimized the contribution of the motion on the GLM residuals. However, we also computed the number of frames with Framewise Displacement (FD)^43^ higher than 0.25 mm. On average, the contribution of frames with high FD was about 1.5% of the total number of frames used to compute the FC and its variability. The standard deviation across subjects was about 2% and the maximum value was 4.4% of the total number of volumes. Using a threshold of 0.5 mm^43^, the maximum FD was about 0.8% of the total number of volumes. Importantly, we computed the partial correlations between FC (and its variability) and CP controlling for the number of frames with high FD. ROI pairs showing a significant correlation between background FC and CP and between FC variability and CP remained significant even when taking the head motion into account.

Overall, these control analyses suggest that sampling variability did not drive our results and that the contribution of motion in the analyzed data was marginal.

### Near-far modulation of FC dynamics in individuals with narrow and wide PPS boundary

Finally, we separated our participants into two groups according to their CP by a median split. One group of participants was characterized by low CP values corresponding to wider PPS (the CPl group). The other group was characterized by high CP values indicating that their PPS was narrower (the CPh group). Specifically, we wanted to test whether FC and the variability of FC differentiated between near and far space in individuals with either wide or narrow PPS, or in both. Mixed ANOVAs showed that only the FC variability can reveal specific differences between near and far processing, while the strength of FC did not. In particular, results showed a significant interaction between the two factors group (CPh, CPl) and space (near, far) only for PM-left IPL (F(1,26) = 9.072, *p* = 0.006) and PM-right FEF (F(1,26) = 5.33, *p* = 0.03) (Fig. 5D, E). Post-hoc comparisons revealed that variability of the FC significantly differed between far and near conditions only in the CPh group for both PM—left IPL (CPh group: *p* = 0.008; CPl group: *p* > 0.05) and PM—right FEF (CPh group: *p* = 0.04; CPl group: *p* > 0.05). As a control, the same analyses conducted on both PM—left IPL and PM—right FEF FC showed no significant effects (*p* = 0.42 and *p* = 0.43, respectively). Hence, the PM—left IPL and PM—right FEF background FC variability is able to capture near-far modulation of space processing. In the specific context of the audio-tactile task adopted in our study, where the maximum distance tested is approximately 98 cm from the body, such near-far modulation is specifically revealed for individuals with narrow PPS boundary.

## Discussion

In this study, we investigated whether and to what extent the individual PPS boundary is associated with the “background” functional connectivity, and its across-trial variability, between the left premotor cortex and other regions differentiating between near and far space. Overall, the strength of the connectivity between PM and other task-relevant nodes was generally higher during the processing of near as compared to far space. Specifically, for the near condition, we found that the individual CP was positively associated with PM—left IPL FC and PM—right dPoCe FC (larger central point implies narrower PPS boundaries). On the other hand, for the far condition, we found a positive relationship between the individual CP and the across-trial FC variability between PM and the following nodes: left IPL, left dPrCe, right FEF, right dPoCe, right vPoCe-SMG.

### Premotor-parietal background connectivity during processing of near space links to individual PPS extension

Our results suggest that the premotor-parietal functional connectivity is associated with the individual PPS extension. This association, observed during near space processing, is specific for the background FC, and independent from any possible contribution by the stimulus evoked activity (as suggested by the beta series correlation analysis run over the background signal). More specifically, the background connectivity pattern associated with near space processing involves PM and right dPoCe in the somatosensory cortex and PM-left IPL. Interestingly, the term peripersonal space was introduced by Rizzolatti and colleagues^27,44^ to highlight the close links between somatosensory (i.e., bodily) and visual/auditory processing exclusively pertaining the near body space. Neurons showing both multisensory and PPS responses have been found in different parietal regions of the monkey brain^45^. Accordingly, recent intracranial electroencephalography studies in humans^46^, which used the same audio-tactile interaction task as the one adopted in the present work, were able to identify electrodes in the right postcentral cortex that showed both audio-tactile multisensory integration and near-far modulation of responses.

Our hypothesis is that multisensory PPS neurons specifically representing the near-body space in both parietal^47–49^ and premotor cortices^27^ of non-human primate might mediate the background connectivity between PM and parietal regions (such as dPoCe and IPL) during the processing of near space, as a function of individual PPS extension: the more the boundary of PPS maps close to the body, the stronger the background connectivity between premotor and parietal neurons that are sensitive to the distance of sensory input from the body. A putative explanation for why narrower PPS is associated with higher connectivity than wider PPS may be referred to the different effects on neuronal discharge that smaller or larger receptive fields, respectively, may exert^50,51^. Importantly, premotor and parietal regions hosting “PPS neurons” seem to be strongly interconnected with each other in both non-human^28,52–55^ and human primates (see the meta-analysis by Grivaz and colleagues^31^). A similar PPS system in humans has been consistently described in several neuropsychological and neuroimaging studies (see^5^ for a recent review), although only at the level of responses.

Despite we stimulated the participants’ right hand, we found that the connectivity of left and right parietal regions with left PM accounts for individual differences in PPS extension: in both cases, the stronger the connectivity, the narrower the PPS. The involvement of the right hemisphere is in agreement with the right-hemisphere dominance model^56^ and, more specifically, with prior evidence of the involvement of right fronto-parietal regions in audiospatial perception^57^, localization of sound sources^58,59^ and reachability estimates of stimuli located within PPS^16^. Notably, the present study remains the first, at our knowledge, that tested the background connectivity between premotor and parietal areas during an implicit PPS task (see^60,61^ for a different approach, based on the resting-state functional connectivity).

### Variability of frontal and parietal background connectivity in participants with narrow PPS

Prior evidence showed that neural variability enables sensorimotor plasticity and dynamic sensorimotor representations^24,25^. In the present work, we analyzed the variability of the background functional connectivity, which reflects the instability of the synchronization between two regions. Inspecting the variability of the background FC between PM and task-relevant DAN, FPN and AN regions, we found a significant positive relationship between the individual CP and the across-trial FC variability b etween PM and left IPL, left dPrCe, right FEF, right dPoCe, right vPoCe-SMG, specifically for the far condition.

Most of these nodes (except left IPL) belong to the DAN, which mediates the capacity to detect and orient toward stimuli in surrounding space^34^ driven by current action goals. This is consistent with the traditional role ascribed to PPS in guiding hand actions toward objects within reaching distance^45,62–65^. In particular, as our results suggest, it may be that less extended representation of PPS—the individual reaching space—are associated to less stable connections between PPS brain regions, such as PM, and regions involved in orienting attention toward the relevant location of target objects, especially in the far space.

Most importantly, we revealed larger variability of PM-left IPL and PM-right FEF background connectivity during far, as compared to near, space processing in individuals with relatively narrow PPS (CPh group).

As for the variability of PM-left IPL background connectivity, we speculate that the near-far modulation may reflect the fluctuations of the signal-to-noise ratio (SNR) in the background activity produced by the different amount of PPS neurons elicited in PM and IPL during the processing of near and far stimuli. It is possible that, in individuals with narrow PPS, when a far auditory stimulus weakly activates only a few multisensory PPS neurons in PM and IPL, the probability for a tactile stimulus to elicit the same neurons is low, thus reducing the SNR in the background signal and consequently increasing the FC variability. Conversely, a near auditory stimulus activates most of the PPS neurons in PM and IPL and the probability for a tactile stimulus to elicit them is higher, with a consequent stronger SNR in the background signal (lower variability). This should not happen in individuals with more extended PPS boundary, for whom the far space becomes near, at least within the spatial distances elicited by looming sounds in this study. In these individuals, less variable PM-IPL background connectivity may similarly support processing of far stimuli, as near stimuli. We hypothesize that this may occur also in participants with narrow PPS after training with a tool leading to an extension of PPS boundary.

As for the variability of PM-FEF background connectivity, it is known that also neurons of FEF are modulated by the distance at which an object is presented^66^. Moreover, lesion studies in non-human and human primates suggest that FEF plays a crucial role for extrapersonal space awareness^67,68^. In fact, they show extrapersonal space neglect after damage to the FEF. Hence, given the role of FEF in linking attention and motor functions^69–72^ for far space processing and awareness, it is not surprising that, as our results show, only participants with narrow PPS show more variable PM-FEF background connectivity supporting far space, as compared to near space processing.

### Methodological considerations on the variability of background connectivity between PM and frontalparietal regions

To estimate the TVFC producing the results presented in this work, we adopted the sliding window approach, which is the most popular method extensively adopted in the literature for resting state and task analyses^73,74^. Using this approach, we evaluated stimulus-specific FC and its variability across trials. We acknowledge that spurious variability generated by sampling variability effects and by the contribution of noise sources (e.g. head motion) might affect our results (but alternative approaches would suffer from similar issues)^39,42,75^.

However, we did not evaluate whether and how much FC varied during a single experimental condition, which triggered the above-mentioned criticism towards the sliding window approach. Rather, we evaluated whether FC variability was significantly different between two experimental conditions (Fig. 5D, E) and was correlated with PPS extension (Fig. 5A–C). Given that sampling variability and noise sources should equally affect each experimental condition, eventual spurious effects are mitigated in our study compared to the analyses of single conditions. Moreover, we performed three control analyses that tested the impact of (i) the window length used for the sliding window computation, (ii) the autocorrelation within time series, and (iii) the head motions on our results. These control analyses confirmed the robustness of our results against possible methodological confounds due to the sliding window approach.

Finally, our results add new insights on the neural underpinnings of individual differences in PPS extension and are unlikely a mere consequence of the previously shown correlation between BOLD signal variability in PM and CP^19^. The correlation between the across-trial FC variability and the trial-by-trial variability is far from being significant. In addition, if the trial-by-trial variability of PM produced the present correlation with CP, one would expect an effect on all the regions of DAN, FPN and AN. Conversely, our effects are spatially selective for a subset of nodes belonging to DAN and FPN (Fig. 5).

## Conclusions

In sum, individuals with narrower PPS show a PM-left IPL and PM-right dPoCe FC that is stronger during the processing of near space. Moreover, the same individuals show a PM-left IPL and PM-right FEF FC that is more variable during processing of far space, as compared to near space. These results are consistent with the notion that dynamic sensorimotor representations are enabled by neural variability^24,25^. Moreover, these results directly point at a key and specific role of the background frontal and parietal connectivity in setting the extent of PPS, at the individual level.

## Materials and methods

### Participants

In the present study, we reanalyzed the fMRI data collected by Ferri et al.^19^. In that study, in the main behavioral experiment, the authors obtained reliable estimation of PPS extension from 28 healthy volunteers (12 females, mean age 21.8 years, range 20–31), who also took part in the fMRI experiment. All the participants were right-handed and took part in the study after providing written informed consent. The experimental protocol was approved by the University G. D’Annunzio of Chieti institutional ethics committee and the research was performed in accordance with relevant guidelines and regulations.

### Behavioral session

#### Task and data acquisition

To estimate the individual PPS boundary, we used a well-established task that has been extensively and successfully used to capture the individual differences in PPS extension and its plasticity (e.g.^9,10,15,19,35,76,77^). Briefly, auditory stimuli, delivered via loudspeakers, were samples of pink noise of 3100 ms duration, either looming or flat. One loudspeaker was placed near to the participants’ right hand and the other at a distance of 100 cm. Looming sounds increased their intensity in time (from 55 to 70 dB) giving the impression of an approaching object. Flat sounds had constant auditory intensity (62.5 dB). They allowed controlling for the impact of auditory intensity changes on audio–tactile interaction. Supra-threshold tactile stimuli were delivered, via pairs of electrodes placed on the index fingers, at five different temporal delays (T1–T5) after the sound onset (Fig. 1A): T1, 300 ms; T2, 800 ms; T3, 1500 ms; T4, 2200 ms; and T5, 2700 ms. Therefore, they occurred when the looming sound, but not the flat sound, source was perceived either close to the body (higher temporal delays) or far from the body (lower temporal delays). To allow the conversion of the temporal dimension of the paradigm into the spatial dimension (i.e. the physical distance of the auditory stimuli), we first identified the exponential function, which better describes our looming sounds. To this aim, we used the formula a * exp(b * x), then we computed the intensity of the sound at each point of the exponential function. Based on these parameters and assuming the speed of sound as constant, we calculated that our five tactile stimuli were delivered when the approaching sound was at 97.7 cm (T1), 95.3 cm (T2), 88.6 cm (T3), 70.5 cm (T4), and 41.7 cm (T5) from the participant’s hand. Each trial, during which either a looming or a flat sound was presented, was followed by an intertrial interval of 1000 ms. Each participant was presented with a random combination of 18 target stimuli for each temporal delay for the looming and flat sounds randomly intermingled with the catch trials. Trials were equally divided into three blocks.

Participants were blindfolded. Participants were asked to respond as fast as possible to the tactile target, when present, by pressing a button with their left index finger, trying to ignore the auditory stimulus. The rationale of the task is that stimuli from different sensory modalities interact more effectively when arise from the same spatial representation^78^. Accordingly, sounds boost tactile RTs when presented close to the body^79–81^. Hence, RTs to tactile stimuli are expected to progressively decrease as the looming sound is perceived to be closer the body. Differently, they should not be significantly modulated by flat sounds.

#### Data analysis and results

To estimate the individual PPS extension, mean RTs to the tactile targets at the different temporal delays (T1–T5) were fitted to a sigmoidal function (see Fig. 1A), as follows: $y\left( x \right) = \frac{{ymin + ymax \cdot e^{{\frac{x - xc}{b}}} }}{{1 + e^{{\frac{x - xc}{b}}} }}$, where x represents the independent variable (timing of touch delivery in milliseconds); y the dependent variable (RT); ymin and ymax the lower and upper saturation levels of the sigmoid, respectively; xc the value of the abscissa at the central point (CP) of the sigmoid (value of x at which $y = \frac{ymin + ymax}{2}$), and b establishes the slope of the sigmoid at the CP.

For each participant, we then took xc, hereafter referred to as the CP of the curve, as an estimation of the individual PPS extension^35^. We ran t-test analyses using OriginLab (http://www.originlab.com) to assess the statistical acceptance (*p* < 0.05) of individual CPs. The average CP was 1491 ± 411 ms ranging from 774 to 2241 ms (Fig. 1A).

The behavioral performance was also used to individually set the main experimental conditions for the fMRI session, i.e. T_NEAR_ and T_FAR_. T_NEAR_ was assigned to the temporal delay associated with their fastest and less variable mean RTs, whereas T_FAR_ was assigned to the temporal delay associated with their slowest and less variable mean RTs.

### fMRI session

#### Task

Auditory and tactile stimuli were the same as in the behavioral session, except that looming and flat sounds were delivered by headphones, Importantly, using a “sound localization task” we previously demonstrated that differences in the experimental setup between the fMRI session and the behavioral session did not affect the perceived distance of the sound sources^19^. Participants were blindfolded and asked to keep their eyes closed. During each trial, either a looming or a flat sound was presented. Along with the auditory stimulation, participants were always presented with a tactile stimulus. The tactile stimulus could be delivered on either their right (experimental trials) or their left (catch trials) index finger and at two different temporal delays (T_NEAR_ and T_FAR_) from the sound onset. Four different experimental conditions resulted from the mixture between the two types of sounds (looming, flat) and tactile temporal delays (T_NEAR_, T_FAR_): LT_NEAR_ = looming sound/T_NEAR_; LT_FAR_ = looming sound/T_FAR_; FT_NEAR_ = flat sound/T_NEAR_; FT_FAR_ = flat sound/T_FAR_. Each trial was followed by a variable intertrial time (Fig. 1B). Participants were asked to press a button with their left middle finger, as fast as possible, to the tactile target only when it was delivered on their left index finger (catch trials), trying to ignore the auditory stimulus. Each participant was presented with a combination of 48 (active) experimental trials for each condition (LT_NEAR_, LT_FAR_, FT_NEAR_, FT_FAR_) intermingled with 20 (passive) catch trials for the same conditions. A pseudorandom stimulus sequence was designed to obtain short periods of consecutive trials of the same condition and type (two or three trials), allowing us to investigate connectivity modulations while subjects processed near or far stimuli for several trials in a row. Importantly, in the sequence, these blocks of consecutive near and far trials were alternated with single different trials to avoid the possible anticipation of the subsequent trials. The 4 conditions (LT_NEAR_, LT_FAR_, FT_NEAR_, FT_FAR_) and the 2 types of trial (experimental, catch) were equally distributed across 8 runs of 6 min each.

#### Image acquisition

A 3 T Philips Achieva scanner (Institute of Advanced Biomedical Technologies, Chieti, Italy) was used to acquire MRI data using a whole-body radiofrequency coil for signal excitation and an eight-channel phased array head coil for signal reception. BOLD contrast images over the entire brain were acquired with a gradient-echo echoplanar sequence [repetition time (TR), 1550 ms; echo time (TE), 30 ms; 29 axial slices with a 0.5 mm gap; slice thickness, 3 mm; in-plane resolution, 3 × 3 mm].

Anatomical images were acquired via a 3D fast-field echo T1- weighted sequence (1 mm isotropic voxel size, TR/TE = 8.1/3.7 ms, flip angle = 8°).

### fMRI data analysis

#### fMRI data preprocessing

Preprocessing steps of functional data were implemented in AFNI^82^ (http://afni.nimh.nih.gov/afni). Functional images were realigned within and across runs to correct for head motion using six-parameter rigid-body realignment. A slice-timing correction was applied to remove differences in acquisition times between slices. The high-resolution anatomical image and the functional images were coregistered and stereotactically normalized to Talairach space^83^. Functional images were spatially smoothed with a 3D Gaussian filter of 6 mm full-width-at-half-maximum.

#### ROI selection

In the present study, we wanted to examine if the background functional connectivity between PM and other task-relevant regions accounts for individual differences in PPS extension. PM coordinates were taken from^19^, where responses in this region were found to depend on whether the sound was perceived as being in the near or far space, applying the following contrast to BOLD data: [LT_NEAR_ − FT_NEAR_]  −  [LT_FAR_ − FT_FAR_]. Then, among the nodes that constitute the FPN and the DAN^32,84^, we selected only those that were actually activated by the PPS task to include in the functional connectivity analyses. Similarly, we selected only task-activated regions from the auditory network (AN^32,84^), which we expected to be recruited during the PPS task because of the auditory nature of the stimuli, hence representing regions that despite task-relevant were not specific for PPS. Finally, we considered all the nodes of the visual network (VIS^32,84^), which we did not expect to be engaged during the PPS task, to be used as a control for the specificity of task-related network connectivity patterns to PM (i.e. FPN-PM, DAN-PM and AN-PM). In order to identify FPN, DAN and AN regions activated by the PPS task to include in the functional connectivity analyses, we checked their overlap with the maps shown in Fig. 2. First, we obtained activation maps LT_NEAR_ or LT_FAR_ (Fig. 2) by submitting the preprocessed fMRI data to a standard GLM analysis, as implemented in AFNI via the SPMG1 basis function. Group-level t-maps were thresholded at *p* = 0.005. To correct for multiple comparisons, we used Monte Carlo simulation as implemented in the AFNI program AlphaSim, yielding a familywise error rate (FWER) at *p* = 0.05^85^. Then, we checked which of the DAN, FPN and AN ROIs, with a radius of 3 mm comprising about 30 voxels on average, overlapped with the LT_NEAR_ or LT_FAR_ maps. The overlap analysis resulted in 13 ROIs belonging to the DAN, 5 ROIs belonging to the FPN and 15 ROIs belonging to the AN.

#### Estimation of functional connectivity and relationship with CP

To estimate the background FC during the audio-tactile task, we first removed the contribution of the evoked signal. At this aim, the BOLD data from the audio-tactile interaction runs for each participant were submitted to regression analyses using a finite impulse response (FIR) model, as implemented in AFNI via the TENTzero basis function. Evoked signals were modeled without a priori assumption of the hemodynamic response shape by a set of 7 functions covering 7 consecutive MR time points, sampled with a time interval of 1.55 s, aligned with the onset of the stimulus. Experimental conditions (LT_NEAR_, LT_FAR_, FT_NEAR_, and FT_FAR_ for experimental and catch trials), false alarms and missed responses were modeled separately. Motion parameters obtained during head motion correction, signal averaged over the lateral ventricles, and signal averaged over a region centered in the white matter were used as additional nuisance regressors. Therefore, the residual dataset minimized the contribution of the transient evoked responses to individual stimuli and reflected the fluctuations in the BOLD response that were not tied to a particular event but were related to the maintenance of the task set. Next, the residuals from this model were filtered using a low-pass filter with a cutoff frequency of 0.17 Hz^37^, averaged across voxels belonging to each ROI and used for the estimation of the background functional connectivity using the Pearson correlation coefficient. Specifically, we estimated the time-varying Pearson coefficient between residual fluctuations of PM region and of all the ROIs previously described over sliding windows lasting 9 MR frames and stepped by 1 TR, according to the formula:

$$r_{XY} \left( t \right) = \frac{{\mathop \sum \nolimits_{i = t}^{t + 9} \left( {X\left( t \right) - \overline{X}} \right)\left( {Y\left( t \right) - \overline{Y}} \right)}}{{\sqrt {\mathop \sum \nolimits_{i = t}^{t + 9} \left( {X\left( t \right) - \overline{X}} \right)^{2} } \sqrt {\mathop \sum \nolimits_{i = t}^{t + 9} \left( {Y\left( t \right) - \overline{Y}} \right)^{2} } }}$$

All the connectivity analyses focused on LT_NEAR_ and LT_FAR_ conditions (hereafter “near” and “far”) only, because (i) individual PPS extension, as indexed by CP, is estimated from RTs to tactile targets associated to looming, but not flat, sounds; (ii) only the neural responses to a “looming” condition have previously revealed a significant association to individual CP^19^.

The near and far FC values were obtained by averaging time-varying correlation values during blocks of multiple trials (see “Task” section) in which the same space condition (near or far, respectively) was presented. These periods were 16 for each experimental condition (near*,* far) and were characterized by average duration of 15.4 TRs. To consider the intrinsic temporal spread of the correlation time course estimated with the sliding window approach, the last 4 points (half window lasting 9 MR frames) of each block were excluded from the average. Then, a Fisher z-transform was applied to the FC averaged across timepoints of the blocks^37^. Importantly, these values are independent of the brain response evoked at every single trial, rather they reflect different “background” brain states associated with specific spatial processing (near and far). This type of approach, successfully used in different cognitive processes^86,87^, has been applied to understand how background functional connectivity supports specific cognitive processes at a broader time scale than individual trials.

First, to examine the specificity of the functional connections between PM and the task-relevant nodes within the DAN, the FPN and the AN (see “ROI selection”), we compared the PM-DAN, the PM-FPN and the PM-AN background functional connectivity with estimations of the connectivity between PM and nodes of the visual network (VIS^84^), which we did not expect to be involved in the processing of audio-tactile stimuli. To this aim, we first averaged FC values over time and across the task-relevant nodes of each network (PM-DAN, PM-FPN, PM-AN), as well as across the nodes of VIS (PM-VIS), regardless of space condition. Then we performed a one-way ANOVA with Network (AN, FPN, VIS) as the within-subject factor on the averaged FC values indexing the time-varying strength of the background functional connectivity to PM. Then, we identified those ROIs showing significant functional connectivity with PM among the task-relevant networks (DAN, FPN, AN), distinctly for each space condition, by performing one-sample t-tests across subjects (*p* < 0.05, Bonferroni corrected).

Finally, to relate the connectivity values obtained between PM and each task-relevant network (DAN, FPN, AN) with individual differences in PPS extension, we calculated the correlation between pairwise FC values (near- and far- specific) and the CP across participants.

#### Variability of functional connectivity and relationship with CP

We estimated the near and far FC variability through the standard deviation of FC values across the sixteen blocks of consecutive near and far trials. Therefore, these values reflect how functional links are more inconstant or stable during task execution.

Then, the FC variability of each ROI pair in both near and far space conditions was correlated with CP across subjects and a permutation test was used to assess the significance of the obtained correlations.

#### ANOVAs on FC and FC variability

Moreover, we separated our participants through a median split into two groups, the *CP low (CPl)* and *CP high (CPh)*, characterized respectively by a wide and a narrow PPS. We performed mixed ANOVA with group (*CPl, CPh*) as the between-subjects factor and space (*near, far*) as the within-subjects factor on the FC and FC variability. Simple effect analyses tested for between- and within-group differences and Duncan correction was used for multiple comparisons.

#### Control analysis on the contribution of the evoked response to FC

To investigate possible significant relationships between individual PPS extension and task evoked activity, we also computed a trial-based measure of functional connectivity using *β*-series correlations^88^. This approach is indeed sensitive to fluctuations in trial-evoked BOLD responses across ROIs. To this aim, for each ROI pair showing a significant correlation between background FC and CP (i.e. PM-left IPL and PM-right dPoCe, see “Results” section), estimates of BOLD responses to individual trials (beta values) were extracted, separately for near- and far-space conditions. The beta values were obtained using a GLM that included separate regressors for each trial and the additional nuisance regressors as in the background FC analysis. This analysis was implemented in AFNI via the SPMG1 basis function. The extracted *β* estimates of each trial were concatenated together in a vector. We then estimated the near and far trial-based FC by correlating the corresponding *β*-series vectors. We also determined whether the trial-based FC was related to individual differences in PPS.

## Data Availability

The empirical data used for this paper are available in the public repository “Mendeley Data” (https://data.mendeley.com/). The codes used for this paper are available upon request from the corresponding author.
